# Supplementary figures and images for: Chronic high-sugar diet in adulthood protects Caenorhabditis elegans from 6-OHDA-induced dopaminergic neurodegeneration
Source: BMC Biol. 2023 Nov 10;21:252. doi: 10.1186/s12915-023-01733-9 (PMC10636816; doi:10.1186/s12915-023-01733-9)

**A**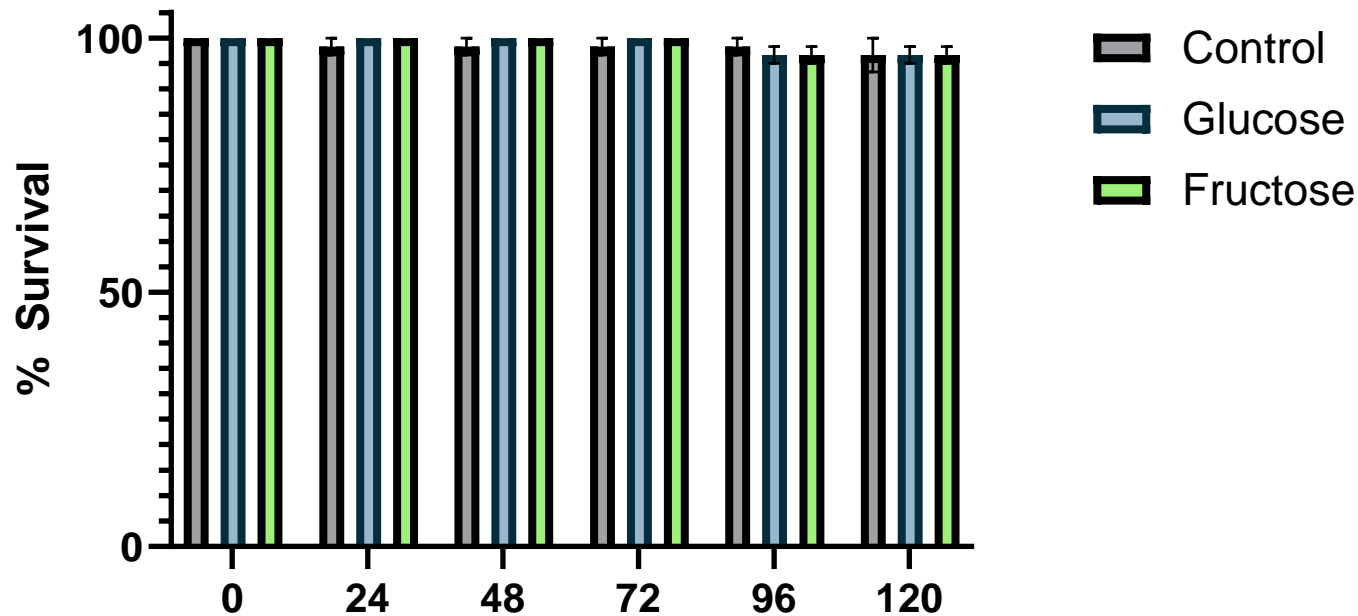

Supplement: Supplementary file 1 — Additional file 1: Fig. S1. Survival of worms during the 5-day exposure to high sugar. BY200 worms were reared to Day 1, at which point 20 worms per treatment per replicate were transferred to control NGM plates or plates containing 100 mM glucose or 100 mM fructose. Each day worms were assessed for survival by touch response with a flame sterilized platinum pick. Surviving worms were transferred to freshly seeded plates each day to avoid plate acidification and to separate them from progeny. For each group 3 biological replicates were assessed, with 20 individuals per replicate. A two-way ANOVA was performed to assess statistical significance, with no differences identified. [file 12915_2023_1733_MOESM1_ESM.pdf]

**Total Mitochondrial Area  
(Fold Change)**

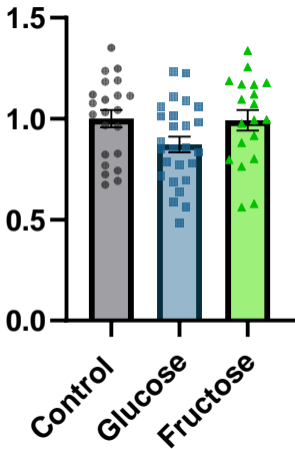

Supplement: Supplementary file 3 — Additional file 3: Fig. S3. Muscle cell mitochondrial mean grey value. SJ4103 worms were reared in accordance with the dietary exposure protocol and imaged on day 8. Images were obtained using a Keyence BZ-X710 with 60X magnification (oil immersion). Z-stacks were set to encompass the entirety of the cell, and maximum projected for analysis. Individual cells were outlined as the region of interest for analysis in Image J. Mean grey value was used as a proxy for total mitochondrial area (One-way ANOVA). [file 12915_2023_1733_MOESM3_ESM.pdf]

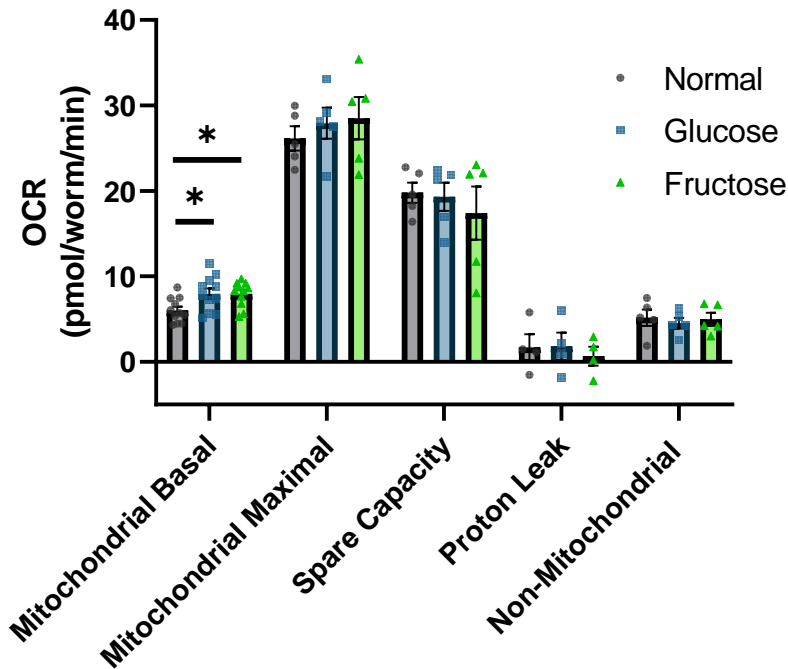

Supplement: Supplementary file 4 — Additional file 4: Fig. S4. Oxygen consumption rate normalized to worm number without accounting for worm size. Whole worm respirometry was performed with the Seahorse XF24 Bioanalyzer and reported as oxygen consumption rate normalized to the number of worms in each well. All wells from the same biological replicate were averaged to produce n=1 (One-way ANOVA, Tukey’s Post Hoc, *p<0.0332). [file 12915_2023_1733_MOESM4_ESM.pdf]

**A**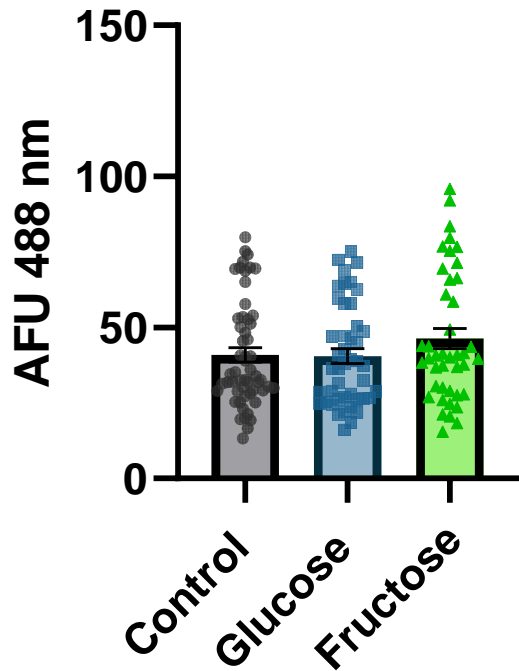**B**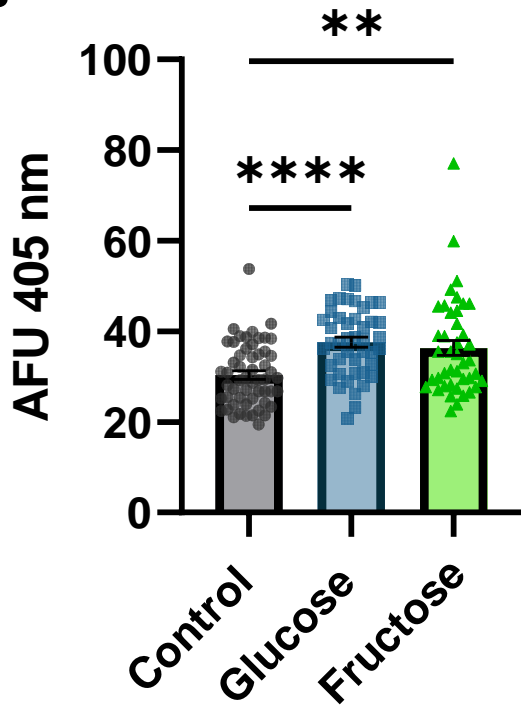

Supplement: Supplementary file 5 — Additional file 5: Fig. S5. Autofluorescence quantification at 488 nm and 405 nm excitation wavelengths. N2 (wild-type) worms were reared on their respective sugar diets and imaged at day 5 of adulthood to determine if high sugar diets alter worm autofluorescence. No significant difference was detected in the N2 strain at 488 nm, however both high glucose and high fructose diets increase autofluorescence at 405 nm excitation (One way ANOVA, Tukey’s Post Hoc,** p<0.0021,**** p<0.0001). [file 12915_2023_1733_MOESM5_ESM.pdf]

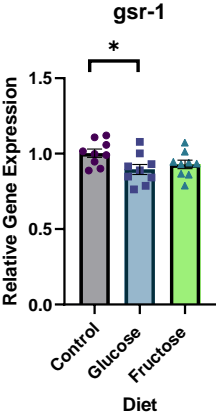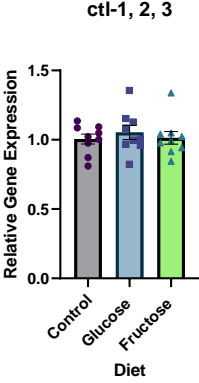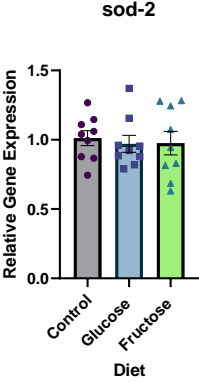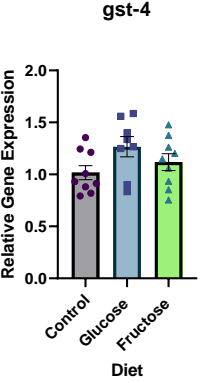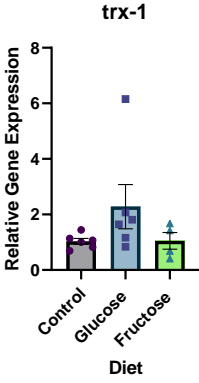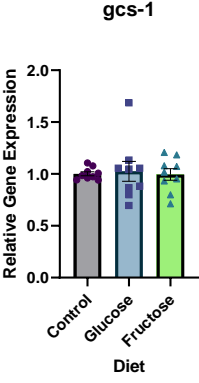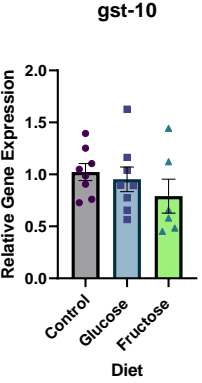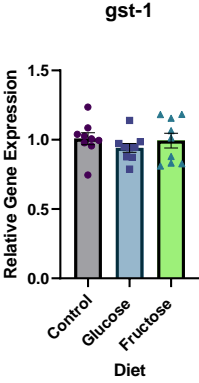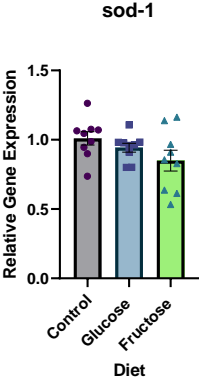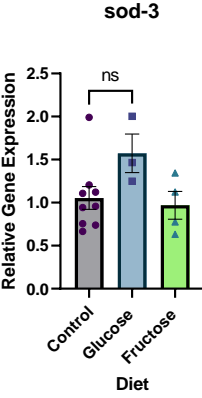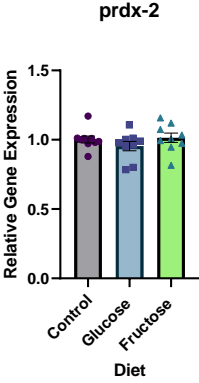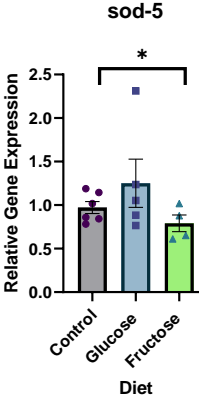

Supplement: Supplementary file 6 — Additional file 6: Fig. S6. Relative gene expression of individual genes. [file 12915_2023_1733_MOESM6_ESM.pdf]

**A**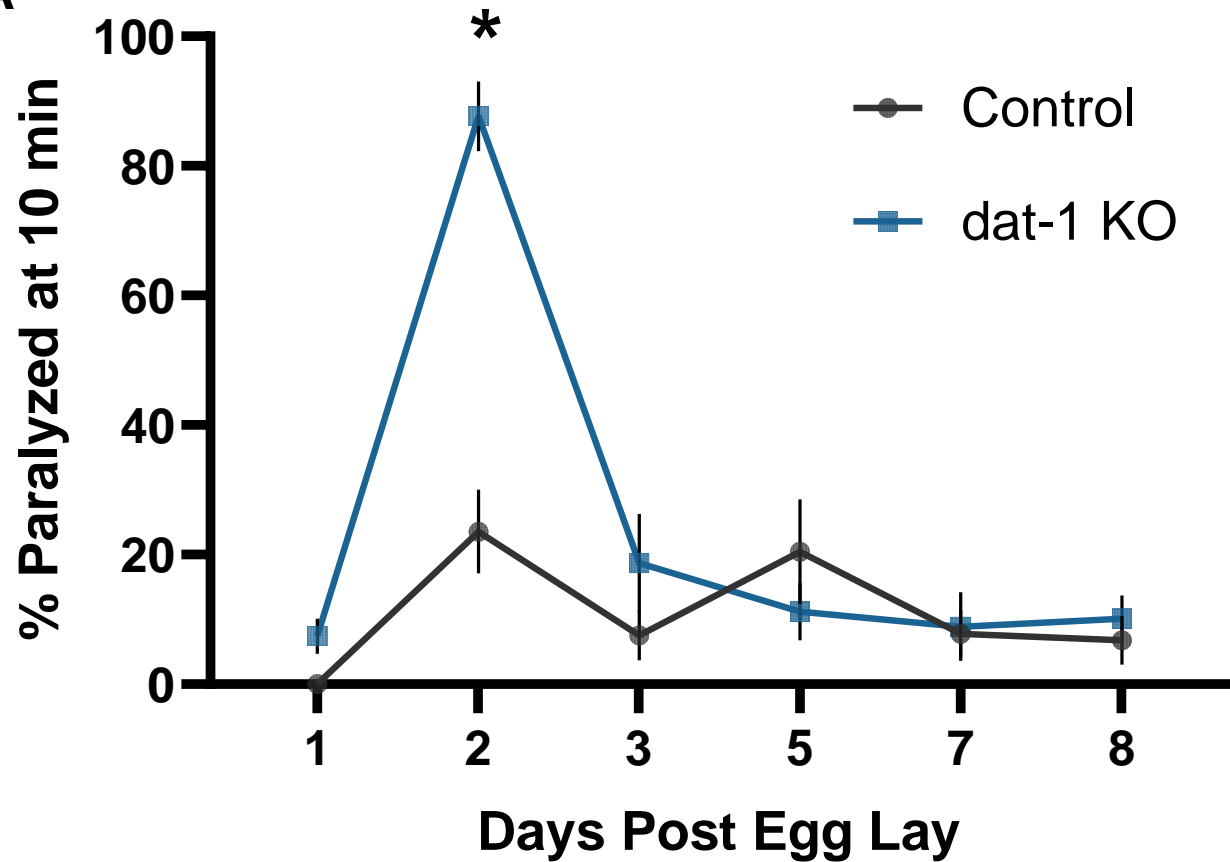

Supplement: Supplementary file 7 — Additional file 7: Fig. S7. Swimming Induced Paralysis Timecourse. BY200 worms were synchronized by timed egg lay and assessed for susceptibility to swimming induced paralysis at times correlating to various developmental and reproductive stages: 24-hours (L2 larval stage), 48-hours (L4 larval stage), 72-hours (early adults), 120-hours (mid-reproductive age adults), 168-hours (late-reproductive age adults), and 192-hours (post-reproductive, experiment timepoint). Three biological replicates were assessed for 24-168 hours, with 2 wells containing approximately 10 individuals each per replicate (n=6 per strain, per timepoint). The data for 192-hours is the data represented for D8 in figure 6D. Results were assessed by two-way ANOVA followed by Sidak’s Test for multiple comparisons with (p<0.05) as the threshold for significance. Only the 48-hour (L4) timepoint indicated a significant difference, p<0.0001. [file 12915_2023_1733_MOESM7_ESM.pdf]
