# Supplementary material for: Chronic high-sugar diet in adulthood protects Caenorhabditis elegans from 6-OHDA-induced dopaminergic neurodegeneration
Source: BMC Biol. 2023 Nov 10;21:252. doi: 10.1186/s12915-023-01733-9 (PMC10636816; doi:10.1186/s12915-023-01733-9)
Supplement: Supplementary file 8 — Additional file 8: Table S1. Primers utilized for RT-qPCR. [file 12915_2023_1733_MOESM8_ESM.docx]

Supplementary Table 1. Primers utilized for RT-qPCR

| Gene | Forward (5’-3’) | Reverse (5’-3’) |
| --- | --- | --- |
| cat-2 | GGCGTTAGAGTTCAAGTTTGGT | CCGCTGTCAAAACCTTCTCC |
| cdc-42 | GAGAAAAATGGGTGCCTGAA | CTCGAGCATTCCTGGATCAT |
| ctl-1,2,3 | ACTAAAGTTTGGCCACACGG | CTTGGAGCATCTTGTCTGGC |
| dat-1 | TTTTGCCATCCGGGTAGAGTC | GACATTGCTCTTTCCCTCTTCG |
| dop-3 | CTTCTTGCTCGCTCTCGTTG | TGGAAGAGAACGATGAATGCG |
| gcs-1 | ACAAGCCGAAGAGCAGGTGAATG | GCAAGCGATGAGACCTCCGTAAG |
| gsr-1 | CGGATTTGATGTGACGCTTA | AAAGTTGCACGTCCTCGAAT |
| gst-1 | CCGTCATCTCGCTCGTCTTAATGG | AGCCTTGCCGTCTTCGTAGTTTC |
| gst-10 | TGGGAAGAGTTCATGGCTTG | AACTTCACTAGAGCCTCCGG |
| gst-4 | AGTTGTTGAACCAGCCCGTGATG | GCCCAAGTCAATGAGTCTCCAACG |
| prdx-2 | TATCGCCTTCTCTGACCGTG | AAGAGTCCACGGAAAGCAATT |
| sod-1 | AAAATGTCGAACCGTGCTGT | CCGGGAGTAAGTCCCTTGAT |
| sod-2 | CTCGCTGCCAGATTTACCAT | TGAACTTGAGAGCTGGCTGA |
| sod-3 | GCAATCTACTGCTCGCACTG | CAGCCTCGTGAAGTTTCTCC |
| sod-5 | AAACGTGCTGTAGCGGTTCT | TCCATGAAGTCCTGGTGACA |
| trx-1 | AGCGGAAGATCTTTGTTCCA | AATTGCGTCTCCATTCTTGG |
| tba-1 | TCATCTCGCAGGTTGTGTCT | GGTAAGCCTTGTCAGCAGAG |
