# Supplementary material for: Chronic high-sugar diet in adulthood protects Caenorhabditis elegans from 6-OHDA-induced dopaminergic neurodegeneration
Source: BMC Biol. 2023 Nov 10;21:252. doi: 10.1186/s12915-023-01733-9 (PMC10636816; doi:10.1186/s12915-023-01733-9)
Supplement: Supplementary file 2 — Additional file 2: Fig. S2. P-values for individual chi-squared tests for analysis of dopaminergic neurodegeneration. Chi-squared tests were used to determine the outcome of 15 comparisons with a Bonferroni corrected p-value of 0.0033. Within each diet, the vehicle was compared to both doses of 6-OHDA. Across diets each treatment was compared. Results are color coded for clarity: grey was not assessed, green is statistically significant, yellow is not statistically significant. [file 12915_2023_1733_MOESM2_ESM.pdf]

Bonferroni corrected p-value: 0.00333		Control			Glucose			Fructose		
		Vehicle	25 mM 6-OHDA	50 mM 6-OHDA	Vehicle	25 mM 6-OHDA	50 mM 6-OHDA	Vehicle	25 mM 6-OHDA	50 mM 6-OHDA
Control	Vehicle		0.0010	<0.0001	0.4706			0.4706		
	25 mM 6-OHDA					0.0027			0.0195	
	50 mM 6-OHDA						<0.0001			<0.0001
Glucose	Vehicle					0.0021	<0.0001	0.4706		
	25 mM 6-OHDA								0.3798	
	50 mM 6-OHDA									0.1120
Fructose	Vehicle								0.0229	<0.0001
	25 mM 6-OHDA									
	50 mM 6-OHDA									
